# Isavuconazole for the Treatment of Fungal Infections: A Real-life Experience From the Fungal Infection Network of Switzerland (FUNGINOS)

**DOI:** 10.1093/ofid/ofae223

**Published:** 2024-04-26

**Authors:** Jade Couchepin, Ilana Reinhold, Ilona Kronig, Monia Guidi, Thierry Buclin, Peter W Schreiber, Dionysios Neofytos, Frederic Lamoth, for the Fungal Infection Network of Switzerland (FUNGINOS), Werner Albrich, Sabina Berezowska, Christoph Berger, Anne Bergeron, Pierre-Yves Bochud, Katia Boggian, Anna Conen, Stéphane Emonet, Véronique Erard, Christian Garzoni, Daniel Goldenberger, Vladimira Hinic, Cedric Hirzel, Nina Khanna, Malte Kohns, Andreas Kronenberg, Frederic Lamoth, Basile Landis, Oscar Marchetti, Konrad Mühlethaler, Linda Müller, Dionysios Neofytos, Michael Osthoff, Jean-Luc Pagani, Chantal Quiblier, Ilana Reinhold, Arnaud Riat, Niels Rupp, Dominique Sanglard, Peter Werner Schreiber, Martin Siegemund, Laura Walti

**Affiliations:** Infectious Diseases Service, Department of Medicine, Lausanne University Hospital and University of Lausanne, Lausanne, Switzerland; Department of Infectious Diseases and Hospital Epidemiology, University Hospital of Zurich and University of Zurich, Zurich, Switzerland; Division of Infectious Diseases, University Hospital of Geneva, Geneva, Switzerland; Service of Clinical Pharmacology, Lausanne University Hospital and University of Lausanne, Lausanne, Switzerland; Institute of Pharmaceutical Sciences of Western Switzerland, University of Geneva, Geneva, Switzerland; Centre for Research and Innovation in Clinical Pharmaceutical Sciences, Lausanne University Hospital and University of Lausanne, Lausanne, Switzerland; Service of Clinical Pharmacology, Lausanne University Hospital and University of Lausanne, Lausanne, Switzerland; Department of Infectious Diseases and Hospital Epidemiology, University Hospital of Zurich and University of Zurich, Zurich, Switzerland; Division of Infectious Diseases, University Hospital of Geneva, Geneva, Switzerland; Infectious Diseases Service, Department of Medicine, Lausanne University Hospital and University of Lausanne, Lausanne, Switzerland; Institute of Microbiology, Lausanne University Hospital and University of Lausanne, Lausanne, Switzerland

**Keywords:** aspergillosis, hepatotoxicity, liver, mucormycosis, triazoles

## Abstract

This analysis of 116 isavuconazole therapy courses shows that hepatic test disturbances (HTDs) were relatively frequent (29% of cases) but rarely led to treatment interruption (5%). Importantly, patients with baseline HTDs, including those attributed to a first-line triazole, did not exhibit a higher risk of subsequent HTD under isavuconazole therapy.

Invasive fungal infections (IFIs) are important causes of mortality among patients who are immunocompromised, such as those with hematologic cancer or long-term immunosuppressive therapies [1, 2]. The limited therapeutic options and their associated toxicities are factors that may contribute to poor outcomes [3, 4]. Triazoles are widely used antifungals for the treatment of IFI because of their large antifungal spectrum and the availability of intravenous and oral formulations [5]. However, their drug-drug interactions and liver toxicity limit their use [5]. Isavuconazole (ISA), the most recently marketed triazole, displays some advantages over other drugs of this class, such as broad antimold activity, fewer drug-drug interactions, and less hepatotoxicity [6]. ISA has been approved for the treatment of invasive aspergillosis (IA) and invasive mucormycosis (IM) [7–10]. It has been available in Switzerland since 2017. This study of the Fungal Infection Network of Switzerland (FUNGINOS) aimed to investigate the current place of ISA for the treatment of IFI in Switzerland, as well as its related outcomes and safety in a real-life clinical setting.

## MATERIALS AND METHODS

Patients treated with ISA between 1 January 2017 and 31 December 2020 were identified via the pharmacology databases in 3 university hospitals (Geneva, Lausanne, and Zurich). Patients having received ≥7 days of ISA therapy with clinical follow-up were included. Demographic characteristics, underlying diseases, characteristics of IFI, type and duration of antifungal therapy, outcomes, and potential adverse events were collected in medical records. When ISA was administered as a second or subsequent therapeutic line, the reason for therapy change was analyzed. Patients were retrospectively followed until the end of ISA therapy or the date of last follow-up and no later than 31 December 2021. IFIs were classified according to criteria of the European Organization for Research and Treatment of Cancer and the Mycoses Study Group Education and Research Consortium [11].

The outcome analysis included only patients fulfilling criteria of proven, probable, or possible IFI. Patients who received ISA as a subsequent line of therapy beyond 28 days from the start of antifungal treatment, which was considered maintenance therapy, were excluded. Response to therapy was assessed at week 6 from the start of ISA therapy. Success was defined as complete or partial response and failure as stable disease, progression, or death, according to standard criteria [12].

For the safety analysis, medical records were screened for adverse events that led to interruption of ISA or were potentially attributed to ISA. Hepatic test values—such as alanine aminotransferase, aspartate aminotransferase, alkaline phosphatase, gamma glutamyl transferase, and total bilirubin—were recorded just before and until the end of ISA therapy. Hepatic test disturbance (HTD) was defined as a ≥2-fold increase from the baseline value of any of the aforementioned liver parameters during ISA therapy.

### Patient Consent Statement

This study was approved by the local ethics committee for retrospective data use (CER-VD, project ID 2020-01641).

## RESULTS

A total of 113 patients who developed 114 IFI episodes and received 116 courses of ISA therapy were included in the analysis. Characteristics of patients and IFI are shown in Table 1. Most patients (81/113, 72%) had hematologic cancer. Criteria of proven/probable or possible IFI were met in 62 (54%) and 37 (32%) of the 114 cases, respectively. In 15 of 114 (13%) cases, there was no IFI criteria. Proven/probable IFI (n = 62) consisted of 40 (65%) IA, 12 (19%) IM, 2 (3%) mixed IA/IM, and 8 (13%) other IFI. Of 116 cases, ISA was administered as first-line therapy in 45 (39%) cases, subsequent therapeutic line in 66 (57%), and prophylaxis in 5 (4%). The median duration of ISA therapy was 97 days (range, 7–953). Among the 68 patients who received ISA as a subsequent therapeutic line (66 as therapy and 2 as prophylaxis), the reasons for switching to ISA were as follows: toxicity attributed to the previous antifungal treatment (n = 33, 49%), failure of the previous antifungal treatment (unsatisfactory response, nonachievement of therapeutic drug monitoring target, or drug-drug interactions; n = 14, 21%), convenience (switch for oral medication; n = 11, 16%), and undetermined (n = 10, 15%).

**Table 1.** Characteristics of Patients, IFIs, and Isavuconazole Therapy Courses

	No (%) or Median (Range)
Patients	113
Age, y	58 (16–83)
Sex	
Female	42 (37)
Male	71 (63)
Underlying conditions	
Allogeneic HSCT	59 (52)
Hematologic cancer, non-HSCT	22 (19)
Solid organ transplantation	12 (11)
Autoimmune disease	3 (3)
Influenza or COVID-19	2 (2)
No host criteria	15 (13)
IFIs^a^	114^a^
IFI classification^b^	
Proven	39 (34)
Probable	23 (20)
Possible	37 (32)
No IFI^c^	15 (13)
Documentation of IFI	62
Invasive aspergillosis^d^	40 (65)
Invasive mucormycosis^e^	12 (19)
Mixed aspergillosis/mucormycosis	2 (3)
Other IFI^f^	8 (13)
Localization of IFI	
Pulmonary only	81 (71)
Extrapulmonary (single site)	10 (9)
Multiple sites	16 (14)
No clinical site	7 (6)
Isavuconazole courses	116^g^
Type of treatment	
First-line therapy	45 (39)
Subsequent line of therapy	66 (57)
Prophylaxis^h^	5 (4)
Isavuconazole monotherapy	100 (86)
Other concomitant antifungal drug^i^	16 (14)

Abbreviations: EORTC-MSGERC, European Organization for Research and Treatment of Cancer–Mycoses Study Group Education and Research Consortium; HSCT, hematopoietic stem cell transplantation; IFI, invasive fungal infection; PCR, polymerase chain reaction.

^a^One patient developed 2 IFIs at 202 days apart.

^b^According to definitions of the EORTC-MSGERC [11].

^c^Includes chronic pulmonary aspergillosis (n = 5), antifungal prophylaxis (n = 5), and suspected IFI not meeting EORTC-MSGERC criteria (n = 5).

^d^Documentation of *Aspergillus* spp (culture or PCR) or positive galactomannan (serum or bronchoalveolar lavage fluid).

^e^Documentation of Mucorales by PCR or culture or presence of broad septate hyphae at histopathology.

^f^Fungal pathogens (n = 1 each): *Scedosporium apiospermum*, *Phaeoacremonium* spp, *Blastoschizomyces capitatus*, *Fusarium* spp, *Conidiobolus* spp, *Trichosporon asahii* and *Kodamaea ohmeri*, *Aureobasidium* spp, positive histopathology only (septate hyphae).

^g^Two patients had 2 distinct courses of isavuconazole therapy for the same IFI episode. The interval between courses was 12 and 29 days, respectively.

^h^First-line prophylaxis (n = 3), second-line prophylaxis (n = 2).

^i^If administered for at least 7 days: echinocandin drug (n = 8), liposomal amphotericin B (n = 7), terbinafine (n = 1).

The response to therapy could be assessed in 62 cases for which ISA was administered as a first line or subsequent therapeutic line within 28 days from the start of antifungal treatment. The success rate for all IFIs was 36 of 62 (58%) at week 6. No significant difference was observed between patients with and without hematologic cancer (63% vs 44%, respectively, *P* = .2). Among proven/probable IFIs, the success rate was 22 of 41 (54%) and did not differ between IA (52%) and other IFI (55%).

For the analysis of adverse events, all 116 ISA therapy courses were included. A total of 34 (29%) patients experienced HTD (Table 2), occurring at a median 20 days (range, 2–318) from the start of ISA therapy. Mild HTD (2- to 5-fold increase of any parameter) and moderate HTD (5- to 10-fold increase) were observed in 26 (76%) and 8 (24%) cases, respectively. Alkaline phosphatase/gamma glutamyl transferase rise was the predominant HTD. Among the 34 patients with HTD, ISA therapy was interrupted in 6 cases (18%) for attributed hepatotoxicity and in 2 cases for other causes (rash, therapy failure). The rate of discontinuation was similar between patients with mild and moderate HTD (23% and 25%, respectively). Considering the entire population, the rate of ISA discontinuation for attributed hepatotoxicity was 6 of 118 (5%). Following ISA interruption, partial resolution of HTD was observed in 4 cases, while 2 patients died from causes not attributed to hepatotoxicity. When ISA therapy was continued despite HTD (n = 26), complete/partial resolution was observed in 15 (58%) cases, while it remained stable in other cases.

**Table 2.** HTDs During Isavuconazole Therapy

	No. (%)
Occurrence of HTD^a^	
All isavuconazole courses (n = 116)	34 (29)
Patients with baseline HTD (n = 58)	18 (31)
Patients without baseline HTD (n = 58)	16 (28)
Patients with previous azole-related HTD (n = 19)^b^	3 (16)
HTD type	34
ALP/GGT only	19 (56)
ALP/GGT and ALT/AST	8 (24)
ALT/AST only	4 (12)
TB only	1 (3)
ALP/GGT and TB	1 (3)
ALP/GGT and ALT/AST and TB	1 (3)
Intervention/outcome	34
ISA interruption: not HTD attributed	2 (6)
ISA interruption: HTD attributed	6 (18)
Improvement^c^	4 (67)
ISA continuation	26 (76)
Improvement^c^	15 (58)

Abbreviations: ALP, alkaline phosphatase; ALT, alanine aminotransferase; AST, aspartate aminotransferase; GGT, gamma glutamyl transferase; HTD, hepatic test disturbance; ISA, isavuconazole; TB, total bilirubin.

^a^Increase ≥2-fold from baseline value of any parameter (ALP, GGT, ALT, AST, TB).

^b^Patients who received isavuconazole as second-line therapy following hepatotoxicity attributed to another azole drug (voriconazole, n = 18; posaconazole, n = 1).

^c^Complete or partial resolution of HTD.

Half of patients (n = 58) received ISA despite baseline HTD (mild in 60% of cases and moderate in 40%). The occurrence of HTD under ISA therapy in these patients did not significantly differ from that in patients without baseline HTD (31% vs 28%, *P* = .8; Table 2). Among patients with baseline HTD attributed to first-line azole therapy (voriconazole or posaconazole, n = 19), 3 (16%) experienced recurrent HTD under subsequent ISA therapy.

Other ISA-attributed adverse events leading to its interruption were skin rash (n = 1) and gastrointestinal disorders (n = 1).

## DISCUSSION

In this multicenter retrospective study, we analyzed the practices of ISA prescription in Switzerland and its related outcomes and toxicity. While the efficacy and safety of ISA for the treatment of IFI have been assessed in randomized controlled trials [8, 9], post hoc surveillance studies are needed to assess the drug profile in real clinical settings that differ from the selected population of clinical trials [13–18].

ISA is currently approved for the treatment of IA and IM [7, 10]. In our study, these IFIs accounted for most cases (87%) where ISA was used as targeted therapy. The success rate for proven/probable IFI was 54%, which is superior to rates reported in prospective trials [8, 9] but similar to those of other real-life observational studies [14, 17]. In addition, ISA was used as empiric therapy for possible IFI. In this setting, ISA represents an alternative to amphotericin B because of its broad spectrum, oral bioavailability, and lower risk of nephrotoxicity. As observed in other settings [15, 17], ISA was mainly used as a second-line therapy in our study (57% of cases). Toxic issues of previous antifungal drugs motivated the switch to ISA in about half of cases. When compared with other antimold triazoles, ISA has fewer drug-drug interactions and a lower risk for hepatotoxicity [6]. In the SECURE trial, ISA was associated with less hepatobiliary disorders when compared with voriconazole [8]. A meta-analysis confirmed the lower rate of ISA-related hepatotoxicity when compared with other antifungals [19]. Similar observations were made when ISA was used as prophylaxis, with a hepatotoxicity-related ISA discontinuation rate of about 5% (lower vs voriconazole or posaconazole) [20–23].

We observed a similarly low rate of hepatotoxicity-related ISA discontinuation in our study, although HTD occurred in about 30% of cases when based on a low cutoff (≥2-fold increase of any hepatic test). At least partial resolution of HTD was observed in more than half of these cases despite ISA continuation, which suggests that their origin was possibly not ISA related. Indeed, causes and mechanisms of HTD under azole therapy are often multiple and complex [24]. Most interesting, half the patients in our study had baseline HTD at the start of ISA therapy. In that sense, our patient population differed from that of previous prospective trials excluding such patients [8, 9]. The main observation of our relatively large cohort (n = 116) was that patients with baseline HTD or previous hepatotoxicity attributed to another triazole were not more susceptible to develop HTD under ISA therapy, which is in line with results of previous smaller studies [13, 15, 25].

In conclusion, this study shows that HTD under ISA therapy, albeit frequent, rarely requires its interruption. In particular, ISA could be safely used in patients with mild/moderate baseline HTD, provided that liver tests are closely monitored. ISA may also be used as second-line therapy in patients who needed to interrupt first-line voriconazole or posaconazole therapy because of hepatotoxicity, as it is associated with a low rate of HTD recurrence in this setting.

## Notes

***Financial support*.** This work was supported by a grant from Pfizer.

## Appendix

Members of the scientific committee of the Fungal Infection Network of Switzerland (FUNGINOS): Werner Albrich (Cantonal Hospital of Sankt Gallen), Sabina Berezowska (Lausanne University Hospital), Christoph Berger (Children Hospital of Zurich), Anne Bergeron (University Hospital of Geneva), Pierre-Yves Bochud (Lausanne University Hospital), Katia Boggian (Cantonal Hospital of Sankt Gallen), Anna Conen (Cantonal Hospital of Aarau), Stéphane Emonet (Hospital of Wallis), Véronique Erard (Hospital of Fribourg), Christian Garzoni (Clinica Luganese Moncucco), Daniel Goldenberger (University Hospital of Basel), Vladimira Hinic (University Hospital of Zurich), Cedric Hirzel (Bern Inselspital), Nina Khanna (University Hospital of Basel), Malte Kohns (Children University Hospital of Basel), Andreas Kronenberg (Bern Inselspital), Frederic Lamoth (Lausanne University Hospital), Basile Landis (University Hospital of Geneva), Oscar Marchetti (Ensemble Hospitalier de La Côte), Konrad Mühlethaler (Bern Inselspital), Linda Müller (Cantonal Hospital of Bellinzona), Dionysios Neofytos (University Hospital of Geneva), Michael Osthoff (University Hospital of Basel), Jean-Luc Pagani (Lausanne University Hospital), Chantal Quiblier (University Hospital of Zurich), Ilana Reinhold (University Hospital of Zurich), Arnaud Riat (University Hospital of Geneva), Niels Rupp (University Hospital of Zurich), Dominique Sanglard (Lausanne University Hospital), Peter Werner Schreiber (University Hospital of Zurich), Martin Siegemund (University Hospital of Basel), Laura Walti (Bern Inselspital).
